# Crystal-Field Properties of Pentalene Ligands in Erbium(III) Sandwich Complexes

**DOI:** 10.1021/acs.organomet.6c00171

**Published:** 2026-07-07

**Authors:** Siddhartha De, Arpan Mondal, F. Geoffrey N. Cloke, Jinkui Tang, Richard A. Layfield

**Affiliations:** † Department of Chemistry, School of Life Sciences, 1948 University of Sussex, Brighton BN1 9RH, U.K.; ‡ School of Chemistry and Chemical Engineering, 47833 Beijing Institute of Technology, Beijing 102488, P. R. China

## Abstract

The influence of pentalene ligands on the crystal-field properties of erbium­(III) has been examined using the monometallic complexes [Er­(η^8^-Pn^†^)_2_]^−^ (**1**) and [(η^8^-Pn^†^)­Er­(η^8^-COT)]^−^ (**2**) (Pn^†^ = [1,4-(^*i*^Pr_3_Si)_2_C_8_H_4_]^2–^, COT = [C_8_H_8_]^2–^), which were synthesized as [K­(2.2.2-crypt)]^+^ salts. Both compounds display magnetic anisotropy consistent with the prolate ion Er^3+^, but do not exhibit slow magnetic relaxation in zero field. Field-induced AC susceptibility measurements show that relaxation is governed by Raman and quantum tunnelling processes, with no evidence for an Orbach mechanism. Multireference ab initio calculations reveal anisotropic ground Kramers doublets with significant transverse components in both **1** and **2**, along with extensive *M*_*J*_ mixing and low-lying excited states that enable efficient relaxation. Structural analysis shows that the pentalene ligands enforce a low-symmetry coordination environment, which is only partially moderated by incorporation of a cyclo-octatetraenyl ligand in **2**. These results indicate that the pentalene ligands impose a local symmetry on erbium­(III) that facilitates transverse anisotropy and effectively precludes single-molecule magnet behavior.

## Graphical abstract

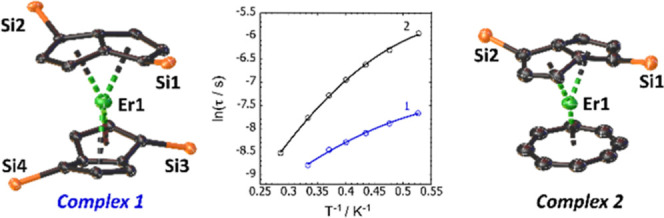

## Introduction

Single-molecule magnets constitute a large and growing family of magnetic materials characterized by their ability to retain magnetization in the absence of an applied field, with this behavior arising from the intrinsic electronic structure of individual metal complexes rather than long-range magnetic order.^1–5^ While the use of SMMs as magnetic storage media has proven challenging to realize in practice, studies of related systems have led to the identification of molecular spin qubits and other functionalities related to quantum information.^6–9^ Organometallic complexes have delivered some of the most prominent lanthanide SMMs, particularly metallocene-based dysprosium and terbium complexes.^10–20^ Many of these systems display exceptional magnetic behavior, including very large effective energy barriers to magnetization reversal and magnetic hysteresis reminiscent of bulk magnets. For oblate spheroidal ions such as Dy^3+^ and Tb^3+^, ‘high-performance’ SMM behavior is typically achieved through a strong axial crystal field that maximizes magnetic anisotropy, combined with minimal equatorial crystal fields that would otherwise promote fast relaxation pathways and restrict the hysteresis.^21^ Dysprosium­(III) complexes with effective two-coordinate, near-linear geometries therefore represent ideal candidates.^22,23^ In a complementary sense, SMM behavior in complexes of the prolate ion Er^3+^ is promoted by dominant equatorial crystal fields with minimal competing axial perturbations, as exemplified by cyclo-octatetraenyl (COT) ligands,^24–40^ although their performance is typically inferior to that observed for SMMs based on oblate lanthanide ions.

Beyond their applications as functional magnetic materials, SMM research has significantly advanced fundamental understanding of crystal-field effects in lanthanide chemistry. Whereas cyclopentadienyl (Cp) and COT ligands have been widely explored, pentalene (Pn) ligands–consisting of two edge-fused cyclopentadienyl rings—remain comparatively unexplored, particularly in terms of their influence on the crystal field of anisotropic lanthanides. Indeed, the sole example of a pentalene-ligated SMM is the dysprosium complex [(η^8^-Pn^†^)­Dy­(Cp*)] (Pn^†^ = [1,4-(^*i*^Pr_3_Si)_2_C_8_H_4_]^2–^, Cp* = [C_5_Me_5_]^−^), which displayed an effective energy barrier of 188(11) cm^–1^ in zero applied field and waist-restricted hysteresis loops up to only 2.4 K, indicative of relaxation via fast quantum tunnelling of the magnetization (QTM).^41^ Multireference calculations revealed that slow relaxation in this SMM arises from a strongly axial crystal field dominated by the Cp* ligand, as reflected in a large negative *B*_2_^0^ parameter and an isolated *M*_*J*_ = ± 15/2 ground state. However, the folded structure of the pentalene ligand introduces substantial transverse terms, notably *B*_2_^2^, which mix multiple excited states and promote QTM. In essence, the crystal-field properties of the Cp* and Pn^†^ ligands work against each other, ultimately limiting the SMM performance.

To further elucidate the crystal-field properties of the pentalene ligand, we now turn to the prolate ion Er^3+^, for which equatorial crystal fields are expected to enhance magnetic anisotropy, thereby providing a complementary test of the crystal-field environment. Our target complexes were the homoleptic *bis*-pentalene complex anion [Er­(η^8^-Pn^†^)_2_]^−^ (**1**) and the heteroleptic COT-ligated complex [(η^8^-Pn^†^)­Er­(η^8^-COT)]^−^ (**2**). Complexes **1** and **2** should allow systematic interrogation of the crystal-field properties of the pentalene ligand, both in isolation and in combination with the COT ligand.

## Results and Discussion

The target complexes **1** and **2** were synthesized as salts of [K­(2.2.2-cryptand)]^+^ via the salt metathesis reactions shown in Scheme 1, with isolated yields of 73% and 79%, respectively. Orange single crystals of both compounds were grown by layering hexane onto concentrated THF solutions at ambient temperature.

**Scheme 1:**
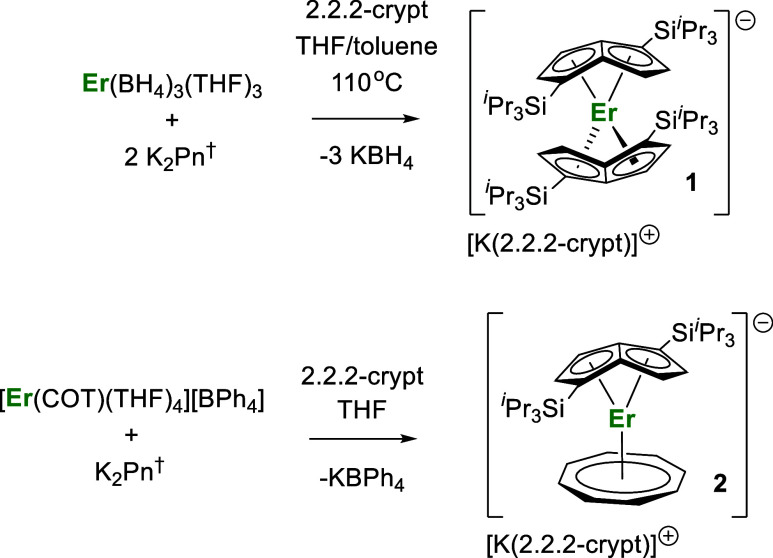
Synthesis of [K­(2.2.2-cryptand)]­[**1**] and [K­(2.2.2-cryptand)]­[**2**]

The structure of the homoleptic complex anion **1** consists of an erbium­(III) center sandwiched between two η^8^-Pn^†^ ligands, with distances to the centroids of the four C_5_ rings in the range 2.281(3)-2.292(2) Å (Figures 1, S3 and S4, and Tables S1–S3). The pentalene ligands adopt a folded structure, with angles of 24.41° and 25.14° formed by intersection of the mean planes of the C_5_ rings. This structural feature produces a broad range of Er–C distances, i.e., 2.392(4)-2.404(4) Å to the body carbon atoms (C2, C3, C10, C11), 2.769(6)- 2.814(5) Å to the wing-tip carbon atoms (C5, C7, C13, C15), and 2.632(4)-2.688(5) Å to the carbon atoms bearing the tri­(isopropyl)­silyl substituents. Considering that the relative orientation of the carbon–carbon bonds shared between the C_5_ ring (i.e., C2–C3 and C10–C11) is not exactly perpendicular, the erbium center in **1** occupies an approximate D_2_-symmetric environment. The structure of **1** is therefore consistent with those of other f-element *bis*-pentalene complexes,^42^ including [Ce­(η^8^-Pn^†^)_2_]^−^,^43^ [M­(η^8^-Pn^†^)_2_] (M = Ce, Th, U),^43–45^ [M­(η^8^-C_8_Me_6_)_2_] (M = Ce, U),^46,47^ and [M­(η^8^-1,3,4,6-C_8_Ph_4_H_2_)_2_]^−^ (M = Y, La, Ce, Tb, Yb).^48^

**Figure 1:**
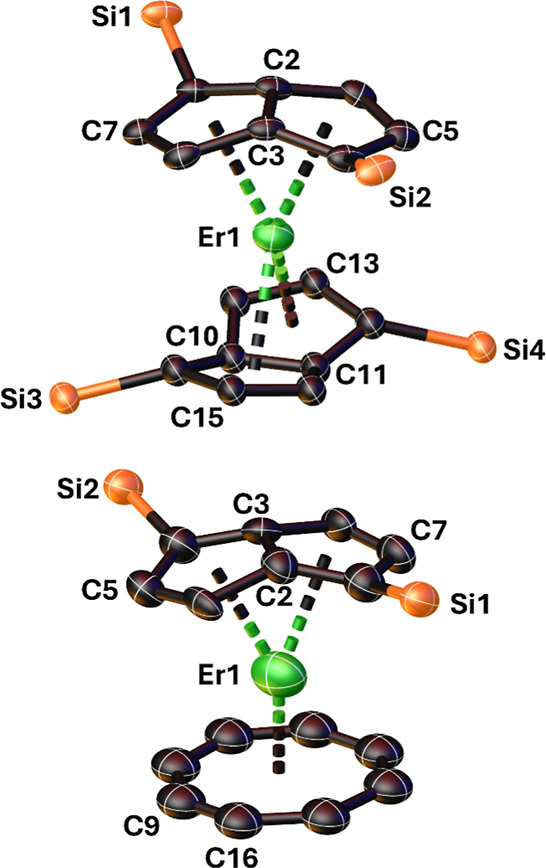
Thermal ellipsoid representations (30% probability) of the complex anions **1** (upper) and **2** (lower). For clarity, the isopropyl substituents and the hydrogen atoms are not shown.

The heteroleptic complex anion **2** features η^8^-bound Pn^†^ and COT ligands, with distances of 2.278(4) Å and 2.288(3) Å from erbium to the centroids of the pentalene ligand and a significantly shorter distance of 1.863(3) Å from erbium to the COT centroid. The distribution of Er–C distances to the Pn^†^ ligand is similar to that in **1**, i.e., 2.386(7) Å and 2.374(8) Å to the body carbon atoms (C2, C3), 2.791(8) Å and 2.799(10) Å to the wing-tip carbons (C5, C7), and 2.632(8)-2.682(9) Å to the other carbon atoms. The pentalene fold angle in **2** is 23.06°. Compared to **1**, the structural impact of swapping a Pn^†^ ligand with COT on the other Pn^†^ ligand in **2** is therefore minimal. Other pertinent structural data are the COT-Er-Pn^†^ angles of 152.06(17)° and 158.25(19)°, and the angle of 172.1(7)° from the midpoint of the C2–C3 bond through the erbium center to the COT centroid, suggesting some axial character of potential relevance to the crystal-field properties.

Although several heteroleptic f-element pentalene complexes with the general composition [(η^8^-Pn^†^)­M­(Cp*)] (M = Y, Dy, U) are known,^41,49^ complex **2** represents the first mixed-sandwich system combining pentalene and COT ligands. It is instructive to compare its structural parameters with those of the related metallole complexes [(η^5^-Cp^E^)­Er­(η^8^-COT)]^−^ (E = Si, Ge, Sn).^36–38^ In these systems, the Er–COT centroid distances are 1.789(4) Å, 1.7610(13) Å, and 1.7653(18) Å, respectively, whereas a significantly longer distance of 1.863(3) Å is observed in **2**. This elongation suggests a reduced interaction between Er^3+^ and the COT ligand in **2**. However, given the presence of the dianionic η^8^-pentalene ligand, which is expected to contribute substantially to the axial and equatorial components of the crystal field, the overall crystal-field environment cannot be inferred from the Er–COT distance alone. Consequently, while the weaker Er–COT interaction in **2** may influence the crystal-field splitting, the balance between axial and equatorial components and, hence, the magnetic anisotropy of the Er^3+^ ion, will depend on the combined effects of both ligands.

### Static-Field Magnetic Properties

Measuring the temperature dependence of the molar magnetic susceptibility (χ_M_) for [K­(2.2.2-cryptand)]­[**1**] and [K­(2.2.2-cryptand)]­[**2**] in an applied DC field of *H* = 1000 Oe revealed behavior typical of a monometallic Er^3+^ complex in both cases (Figures S5–S6). For [K­(2.2.2-cryptand)]­[**1**], the value of χ_M_ *T* at 300 K is 11.07 cm^3^ K mol^–1^, which is close to the theoretical value of 11.48 cm^3^ K mol^–1^ for an Er^3+^ ion with a 4f^11^ configuration and a ^4^I_15/2_ ground term. Upon lowering the temperature, χ_M_ *T* progressively decreases to reach 6.19 cm^3^ K mol^–1^ at 2 K. Similarly, the value of χ_M_ *T* for [K­(2.2.2-cryptand)]­[**2**] is 11.01 cm^3^ K mol^–1^ at 300 K and 4.21 cm^3^ K mol^–1^ at 2 K. The isothermal field-dependence of the magnetization for [K­(2.2.2-cryptand)]­[**1**] at 2 K shows a sharp increase as the field increases to 10 kOe, followed by a more gradual increase up to 70 kOe, where a magnetization value of 4.33 μ_B_ is reached. Comparable *M*(*H*) behavior was found for [K­(2.2.2-cryptand)]­[**2**], with a magnetization of 4.04 μ_B_ at 70 kOe. The magnetization for both compounds at the upper field limit of the magnetometer is considerably smaller than the theoretical saturation magnetization value of 9 μ_B_ for a Er^3+^ free ion, indicating significant magnetic anisotropy in both cases.

### Dynamic Magnetic Properties

The real (χ′) and imaginary (χ″) components of the AC magnetic susceptibility of [K­(2.2.2-cryptand)]­[**1**] and [K­(2.2.2-cryptand)]­[**2**] were studied in an oscillating field of *H*_ac_ = 3 Oe and AC frequencies in the range ν = 1–1488 Hz (Figures 2, S7 and S8). Maxima were not observed in zero DC field for either compound, hence an applied field of 600 Oe was used to reduce the effects of QTM. Field-induced slow magnetic relaxation was observed for [K­(2.2.2-cryptand)]­[**1**] with maxima in the χ″(ν) data observed in the temperature range 1.9–3.0 K, and in the range 1.9–3.5 K for [K­(2.2.2-cryptand)]­[**2**] (Figure 2). Cole–Cole plots of χ″ versus χ′ display depressed semicircular profiles (Figures S9 and S10), which were fitted using eqs S1 and S2 with α-parameters spanning 0.08–0.24 and 0.07–0.44 for [K­(2.2.2-cryptand)]­[**1**] and [K­(2.2.2-cryptand)]­[**2**], respectively. This analysis is consistent with a distribution of relaxation times (τ) in each compound. Plots of ln τ against *T* ^–1^ showed curvature across the full temperature ranges, indicating non-Orbach relaxation. Fits of the data were obtained using the expression τ^–1^ = CT^*n*^ + τ_QTM_^–1^, where *C* and *n* represent the Raman coefficient and Raman exponent, respectively, and τ_QTM_^–1^ is the rate of QTM. This analysis produced *C* = 179 ± 74.61 s^–1^ K^–*n*^, *n* = 3.13 ± 0.34, and τ_QTM_ = 1.2 ± 0.281 ms for [K­(2.2.2-cryptand)]­[**1**] and *C* = 8.44 ± 0.927 s^–1^ K^–*n*^, *n* = 5.07 ± 0.094, and τ_QTM_ = 5.8 ± 0.135 ms for [K­(2.2.2-cryptand)]­[**2**]. Including an Orbach term and effective energy barrier (*U*_eff_) did not improve the quality of the fits.

**Figure 2:**
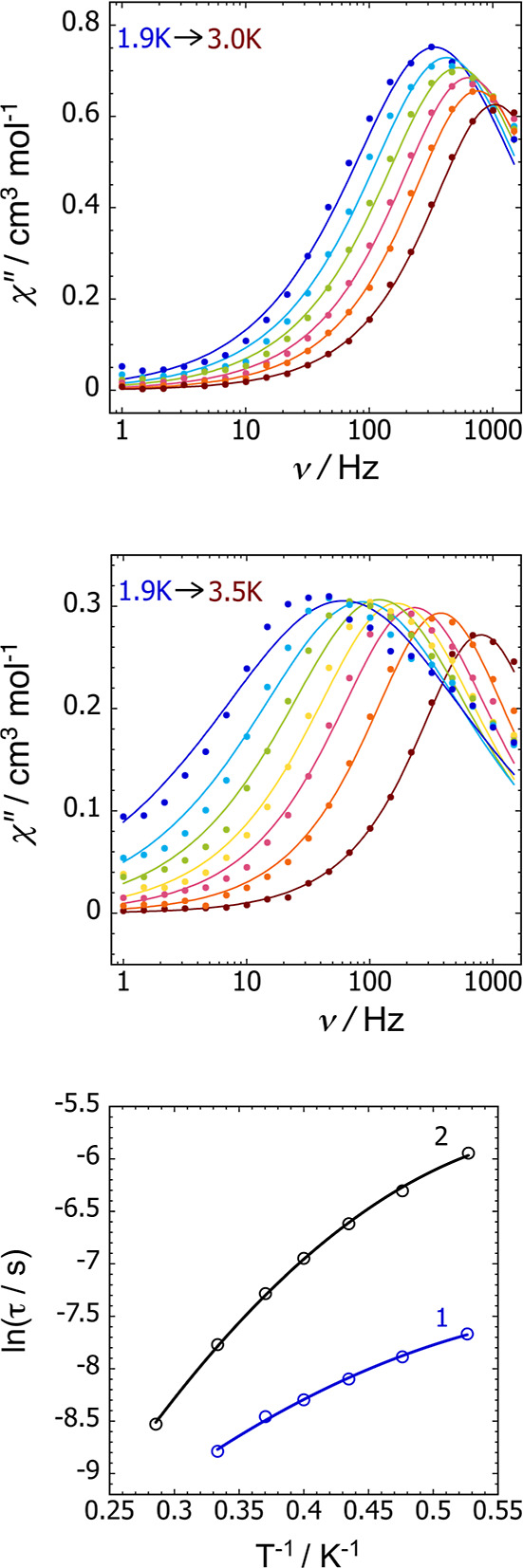
Plots of χ″(ν) for [K­(2.2.2-cryptand)]­[**1**] (top) and [K­(2.2.2-cryptand)]­[**2**] (middle) in an applied field of 600 Oe. Plots of ln τ vs *T* ^–1^ (bottom).

The larger *C* value in **1** and the associated lower Raman exponent indicate more efficient phonon-mediated relaxation in the homoleptic complex, consistent with enhanced spin-phonon coupling.^50,51^ In contrast, **2** exhibits a more typical Raman exponent but still lacks an Orbach relaxation pathway. The corresponding QTM times for **1** and **2** further indicate that tunnelling remains an efficient relaxation pathway in both systems. In agreement with the efficient QTM, neither compound shows open magnetic hysteresis loops (Figures S12 and S13). The absence of slow magnetic relaxation in zero field, together with relaxation dominated by Raman and quantum tunnelling processes and no observable Orbach regime, indicates that both erbium complexes possess crystal-field environments that do not effectively suppress quantum tunnelling. In **1** and **2**, the η^8^-pentalene ligands are likely to generate a low-symmetry crystal field with significant transverse components, resulting in a nonideal environment for the prolate Er^3+^ ion. Consequently, and despite the presence of an equatorial COT ligand in **2**, neither coordination environment can stabilize a well-isolated ground doublet. This situation contrasts with the related axially symmetrical sandwich complex [Er­(η^8^-COT)_2_]^−^ and the C_3v_-symmetric complex Er­{N­(SiMe_3_)_2_}_3_, both of which feature dominant equatorial crystal fields with relatively minor axial components.^25,26,52^ To gain further insight into the magnetic properties of both erbium complexes, multireference calculations of the relaxation pathways and the crystal-field parameters were undertaken.

### Theoretical Studies

Calculations of the CASSCF/QDPT/SINGLE_ANISO type were performed on **1** and **2** using atomic coordinates obtained from the X-ray structures, with hydrogen atom positions optimized using density functional theory. The ORCA 6.0.0 software package was used for all calculations.^53,54^ The calculated *g*-tensors for the ground Kramers doublets (KDs) in **1** and **2** reveal strong anisotropy, with *g*_*z*_ values of 13.86 and 12.75, respectively (Tables S6 and S7). The main magnetic axis in the ground KD for each complex is oriented approximately perpendicular to the molecular symmetry axis (Figure 3). However, both ground KDs feature significant transverse components, as reflected in *g*-tensors of *g*_*x*_ = 0.93 and *g*_*y*_ = 4.30 for **1**, and *g*_*x*_ = 1.65 and *g*_*y*_ = 1.92 for **2**, which compare to the ideal values for an erbium SMM of *g*_*z*_ = 18 and *g*_*x*_ = *g*_*y*_ = 0.

**Figure 3:**
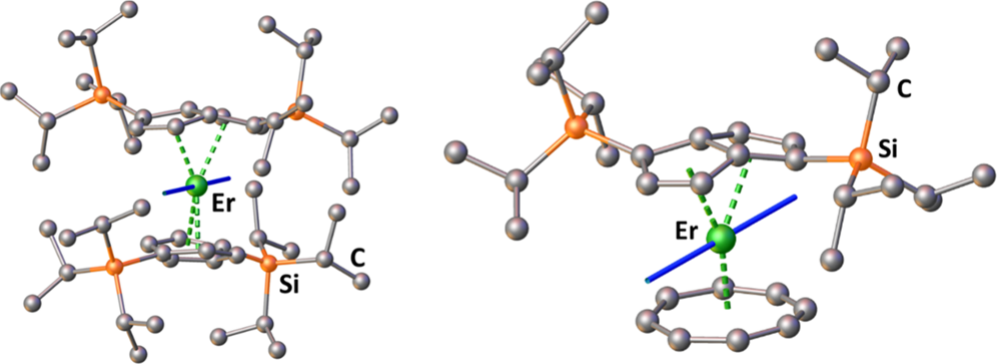
Easy axis of magnetization (blue line) in the ground Kramers doublet of **1** (left) and **2** (right).

The substantial transverse components and appreciable wave function mixing agree with the calculated crystal field parameters for the two complexes (Table S8). The axial crystal-field parameter *B*_2_^0^ differs in sign for **1** and **2**, being −0.23 and 0.21, respectively, indicating dominant axial and equatorial contributions, respectively. For the prolate Er^3+^ ion, a positive *B*_2_^0^ is generally conducive to SMM behavior. However, in both systems the presence of significant nonaxial crystal-field terms *B*_2_^*q* ≠ 0^ dominate, resulting in strong transverse anisotropy and efficient quantum tunnelling. Furthermore, the wave-function compositions for the ground KDs in **1** and **2** are highly mixed, consisting of only 52% |*M*_J_| ≈ 15/2 character, with appreciable contributions from several other wave functions (Tables S6 and S7). The substantial *M*_*J*_ mixing in the two erbium complexes is likely to be responsible for the efficient QTM and absence of observable Orbach regime in both cases. The first-excited KDs in **1** at 45 cm^–1^ and in **2** at 25 cm^–1^ are also strong admixtures of wave functions, and the calculated values of the transition matrix elements between ground and excited KDs are appreciable (Tables S9 and S10). These results indicate multiple efficient relaxation pathways arising from strong coupling between states, reflecting the QTM and Raman processes identified from the AC susceptibility data (Figure 4).

**Figure 4:**
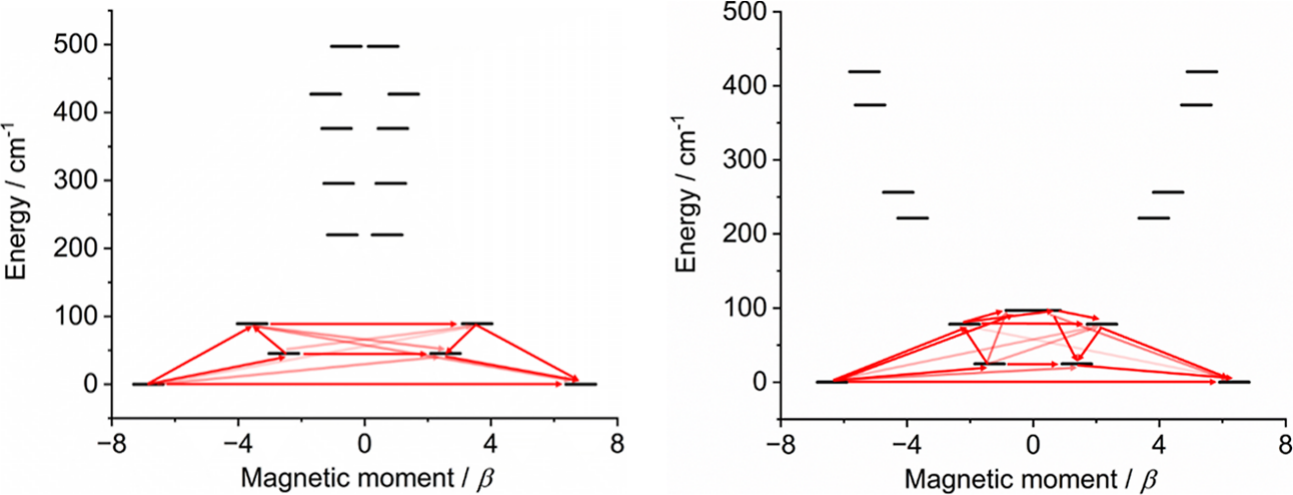
Calculated relaxation barrier for **1** (left) and **2** (right). Red arrows correspond to potential relaxation pathways, with darker shading indicating larger absolute values of the transition magnetic moment matrix elements between the respective states. Transitions involving higher-energy states not involved in the relaxation mechanism are omitted for clarity.

Overall, the η^8^-pentalene ligands in **1** and **2** act as electronically unsymmetrical donors, delivering a low-symmetry crystal field at the Er^3+^ centers. In the *bis*-pentalene complex **1**, the presence of two such ligands magnifies the effect, generating an environment with appreciable axial and equatorial contributions. In contrast, the η^8^-COT ligand in **2** provides a more uniform equatorial crystal field, but its influence is diminished by the elongated Er-COT interaction and the competing contribution of the pentalene ligand. As a result, both complexes exhibit crystal fields that are poorly balanced, with pentalene dominating the electronic structure and preventing the establishment of a symmetry-controlled coordination environment.

## Conclusions

The homoleptic *bis*-pentalene complex [Er­(η^8^-Pn^†^)_2_]^−^ (**1**) and the heteroleptic complex [(η^8^-Pn^†^)­Er­(η^8^-COT)]^−^ (**2**) have been synthesized as their [K­(2.2.2-cryptand)]^+^ salts and structurally characterized, providing a platform to evaluate the crystal-field properties of the pentalene ligand with erbium­(III). Magnetic measurements show that both complexes exhibit significant anisotropy but do not display slow magnetic relaxation in zero field, with relaxation dynamics dominated by Raman and quantum tunnelling processes and no observable Orbach regime. These results indicate that neither ligand environment can stabilize a well-isolated ground Kramers doublet. The magnetic properties can be traced to the structural and electronic characteristics of the ligand environments, particularly the η^8^-pentalene ligand and the associated low-symmetry crystal field at the metal center. In the *bis*-pentalene complex **1**, the presence of two such ligands amplifies this effect, leading to an unbalanced coordination environment. In **2**, the COT ligand introduces a more symmetrical equatorial donor framework, but this is effectively outcompeted by the pentalene ligand. Consequently, both complexes exhibit crystal fields that are insufficiently symmetrical to suppress transverse anisotropy. Overall, these results further highlight the importance of controlling crystal-field strength and symmetry in the design of SMMs based on prolate lanthanide ions.

## Supplementary Material



## Notes

General considerations and synthesis details; X-ray crystallography; IR spectra; NMR spectra; Magnetic property measurements. Other data that support the findings of this study are openly available at DOI: 10.25377/sussex.32253495 (PDF)
