# Supplementary material for: Crystal-Field Properties of Pentalene Ligands in Erbium(III) Sandwich Complexes
Source: Organometallics. 2026 Jul 7;45(14):1740–5. doi: 10.1021/acs.organomet.6c00171 (PMC13418196; doi:10.1021/acs.organomet.6c00171)
Supplement: Supplementary file 1 [file om6c00171_si_001.pdf]

Crystal-Field Properties of Pentalene Ligands in Erbium(III) Sandwich Complexes

Siddhartha De,[a] Arpan Mondal,[a] F. Geoffrey N. Cloke,[a] Jinkui Tang,[b] Richard A Layfield*[a]

[a] Department of Chemistry, School of Life Sciences, University of Sussex, Brighton, BN1 9QR, U.K. E-mail: r.layfield@sussex.ac.uk

[b] School of Chemistry and Chemical Engineering, Beijing Institute of Technology, Beijing 102488, P. R. China.

Contents

General Considerations and Synthesis Details pages S1-S2
FTIR Spectra pages S2-S3
X-ray Crystallography pages S4-S8
Magnetic Property Measurements pages S9-S14
Computational Section pages S15-S19
References pages S20

General Considerations

Experiments were carried out under rigorous anhydrous and anaerobic conditions using standard Schlenk line techniques and argon-filled gloveboxes. Solvents were refluxed over suitable drying agent for a minimum of three days (molten potassium for toluene, THF and Na/K alloy for hexane) and then distilled and degassed via a minimum of three freeze-pump-thaw cycles, and stored in ampoules over potassium mirrors (for toluene and hexane) and activated 4 Å molecular sieves (THF). $K_2Pn^†$ ($Pn^†$ = $[1,4\text{-}(^iPr_3Si)_2C_8H_4]^{2-}$),[1] $[Er(BH_4)_3(THF)_3]$,[2] and $[Er(COT)(THF)_4][BPh_4]$[3] were prepared according to literature procedures. 2.2.2-Cryptand was purchased from Sigma-Aldrich and dried under vacuum at 60 °C for three days before use. All other chemicals were obtained from commercial sources and used without further purification.

Elemental analyses were carried out at Elemental Microanalysis Limited, Devon, United Kingdom. Attenuated total reflectance Fourier-transform infrared spectroscopy (ATR-FTIR) spectra were collected using a Bruker ALPHA spectrometer equipped with a Platinum ATR module in an argon- filled glovebox.

Synthesis Details

Synthesis of [K(2.2.2-crypt)][Er(η^8-Pn†)$_2$] ([K(2.2.2-crypt)][1])

THF (6 ml) was added to a mixture containing $K_2Pn^†$ (50 mg, 1.0 mmol), and $[Er(BH_4)_3(THF)_3]$ (21 mg, 0.05 mmol) at ambient temperature. After stirring overnight at room temperature, 2.2.2-cryptand (19 mg, 0.05 mmol) was added and the mixture was stirred for 6 h. The resulting mixture was evaporated to dryness, and the residue was extracted into THF. Layering the THF extract with hexane at room temperature produced orange crystals suitable for X-ray diffraction after five days (52 mg, 73%). Elemental analysis calculated for $C_{70}H_{128}ErKN_2O_6Si_4$: C, 59.52; H, 9.13; N, 1.98. Found: C, 59.89; H, 8.98; N, 1.95.

Synthesis of [K(2.2.2-crypt)][(η^8-Pn†)Er(η^8-COT)] ([K(2.2.2-crypt)][2])

A 1:1 THF-toluene mixture (6 ml) was added to an ampoule containing $K_2Pn^†$ (25 mg, 0.05 mmol), and $[Er(COT)(THF)_4][BPh_4]$ (44 mg, 0.05 mmol) at room temperature. After brief stirring, the mixture was heated for 12 hours at 110 °C. Then the mixture was cooled to ambient temperature, filtered, and the filtrate was evaporated to dryness. The resulting solid was dissolved in THF (4 mL) and 2.2.2-cryptand (19 mg, 0.05 mmol) was added. The resulting orange-red solution was stirred for 4 h at ambient temperature. The orange-red solution was filtered, and the filtrate was layered with hexane. Storage at ambient temperature produced

orange crystals suitable for X-ray diffraction after three days (44 mg, 79%). Elemental analysis calculated for $C_{52}H_{90}ErKN_2O_6Si_2$: C, 56.69; H, 8.23; N, 2.54. Found: C, 56.25; H, 8.20; N, 2.47.

FTIR Spectra

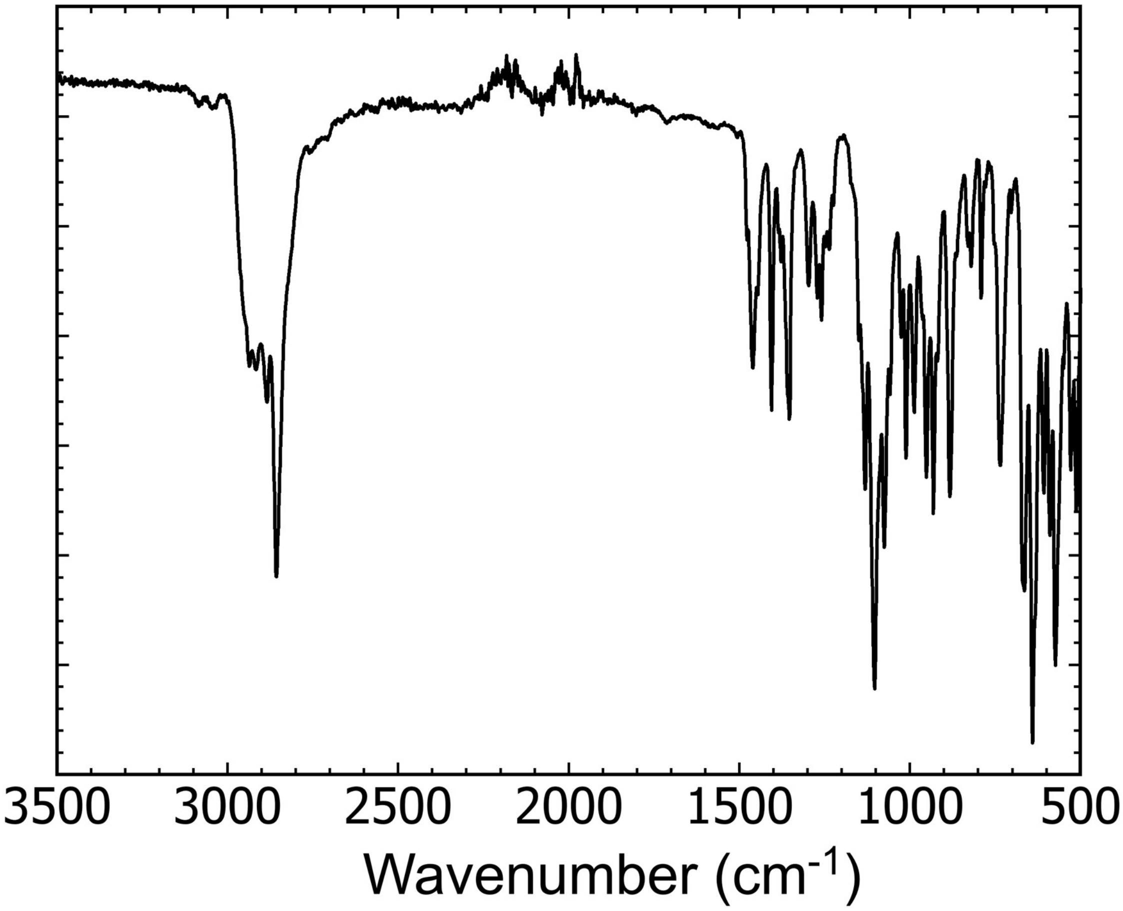

Figure S1. FTIR spectrum of [K(2.2.2-crypt)][**1**]. Major absorptions (cm^{-1}): 2936 (w), 2915(w), 2883(w), 2855 (vs), 1460 (s), 1405 (s), 1355 (s), 1296 (w), 1270 (w), 1258 (w), 1238 (w), 1131 (m), 1104 (vs), 1075 (m), 1025 (w), 1011 (m), 988 (m), 951 (m), 930 (m), 882 (s), 821 (w), 790 (w), 736 (vs), 702 (w), 667 (s), 640 (s), 608 (m), 589 (m), 573 (s), 528 (m), 512 (m), 467 (m), 412 (m).

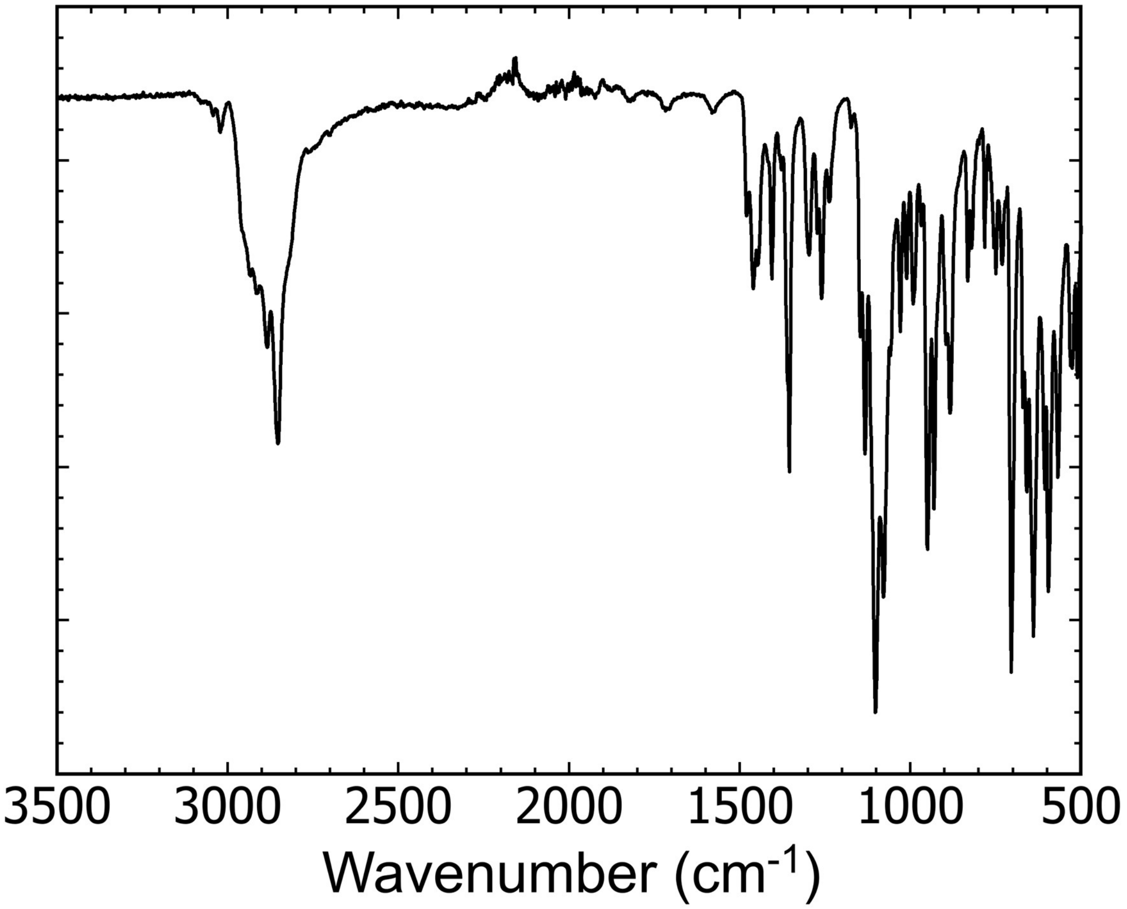

Figure S2. FTIR spectrum of [K(2.2.2-crypt)][**2**]. 3020 (w), 2934(w), 2913(w), 2885 (w), 2853 (vs), 1827 (vw), 1717(vw), 1577 (vw), 1478 (w), 1459 (s), 1405 (s), 1356 (s), 1297 (s), 1271 (w), 1259 (s), 1238 (w), 1145(w), 1132 (s), 1102 (vs), 1079 (s), 1029 (m), 1011 (m), 990 (m), 949 (s), 930 (s), 883 (s), 830 (w), 820 (w), 781 (w), 752 (w), 732 (w), 703 (vs), 668 (w), 658 (m), 640 (s), 603(w), 594 (s), 567 (m), 528 (m), 511 (m), 472 (m), 457 (m), 406(m).

X-ray Crystallography

Single crystals of both compounds were immersed in NVH oil (degassed and dried) and mounted on a Rigaku FR-007HF rotating anode equipped with a Saturn 724+ CCD area detector and a quarter-chi goniometer. Cu-K_{α} radiation (λ = 1.54184 Å) was used. Using Olex2,[4] structures were solved with the olex2.solve[5] structure solution program using charge flipping and refined with the SHELXL[6] refinement package using least-squares minimization. Crystallographic data were deposited at Cambridge Crystallographic Data Centre.

Table S1. Crystallographic data of [K(2.2.2-crypt)][**1**] and [K(2.2.2-crypt)][**2**].

	**[K(2.2.2-crypt)][1]**	**[K(2.2.2-crypt)][2]**
CCDC Number	2464809	2464808
Empirical formula	$C_{70}H_{128}ErKN_2O_6Si_4$	$C_{52}H_{90}ErKN_2O_6Si_2$
Formula weight (g/mol)	1412.45	1101.83
Temperature/K	100.0	100.0
Crystal system	monoclinic	triclinic
Space group	$P2_1/n$	$P\bar{1}$
a/Å	17.16240(10)	12.7778(6)
b/Å	24.2451(2)	15.2865(8)
c/Å	18.69200(10)	16.4172(9)
α/°	90	109.680(5)
β/°	97.1770(10)	106.473(4)
γ/°	90	100.836(4)
V/Å^3	7716.88(9)	2749.1(3)
Z	4	2
ρ_{calc}/g cm^{-3}	1.215	1.329
μ/mm^{-1}	3.448	4.287
F(000)	3008.0	1140.1
Crystal size/mm^3	0.25 × 0.2 × 0.15	0.14 × 0.07 × 0.05
2θ range for data collection/°	6 to 134.198	6.16 to 135.26
Index ranges	-15 ≤ h ≤ 20, -27 ≤ k ≤ 28, -21 ≤ l ≤ 22	-15 ≤ h ≤ 14, -18 ≤ k ≤ 18, -19 ≤ l ≤ 19
Reflections collected	71984	48839
Independent reflections	13635 [R_{int} = 0.0583, R_{sigma} = 0.0279]	9710 [R_{int} = 0.2186, R_{sigma} = 0.1433]
Data/restraints/parameters	13635/78/817	9710/67/511
Goodness-of-fit on F^2	1.025	0.974
Final R indexes [I>=2σ (I)]	R_1 = 0.0560, wR_2 = 0.1452	R_1 = 0.0820, wR_2 = 0.2050
Final R indexes [all data]	R_1 = 0.0600, wR_2 = 0.1490	R_1 = 0.1264, wR_2 = 0.2404
Largest diff. peak/hole/e Å^{-3}	1.62/-1.01	1.76/-2.04

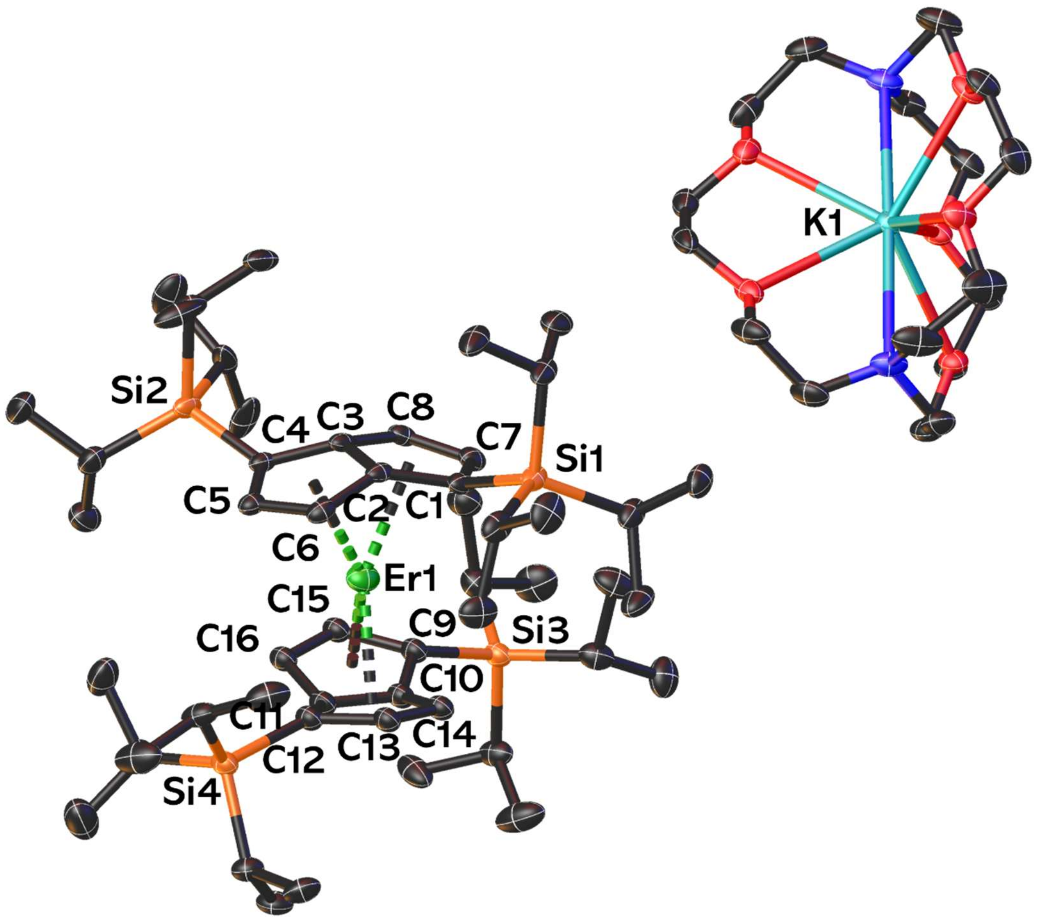

Figure S3. Thermal ellipsoid representation (30% probability) of the structure of [K(2.2.2-crypt)][**1**]. For clarity, hydrogen atoms and one part of the disordered ^{i}Pr groups are not shown.

Table S2. Selected distances and angles for [K(2.2.2-crypt)][**1**].

	**Length/Å**	**Angle/°**
Er1-C1	2.687(4)	
Er1-C2 (br)	2.392(4)	
Er1-C3 (br)	2.404(4)	
Er1-C4	2.687(4)	
Er1-C5 tip	2.788(4)	
Er1-C6	2.632(4)	
Er1-C7 tip	2.814(5)	
Er1-C8	2.661(4)	
Er1-C9	2.678(5)	
Er1-C10(br)	2.392(6)	
Er1-C11(br)	2.393(6)	
Er1-C12	2.688(5)	
Er1-C13 tip	2.769(6)	
Er1-C14	2.639(6)	
Er1-C15 tip	2.786(5)	
Er1-C16	2.642(5)	
C2(br)-C3(br)	1.456(5)	
C10(br)-C11(br)	1.403(6)	
Er1- $Pn^{\dagger}_{cent}$(1)	2.292(2)	
Er1- $Pn^{\dagger}_{cent}$(2)	2.2810(18)	
Er1- $Pn^{\dagger}_{cent}$(3)	2.286(2)	
Er1- $Pn^{\dagger}_{cent}$(4)	2.281(3)	
$Pn^{\dagger}_{cent}$(1)-Er1- $Pn^{\dagger}_{cent}$(3)		142.81(8)
$Pn^{\dagger}_{cent}$(1)-Er1- $Pn^{\dagger}_{cent}$(4)		150.85(7)
$Pn^{\dagger}_{cent}$(2)-Er1- $Pn^{\dagger}_{cent}$(3)		149.33(7)
$Pn^{\dagger}_{cent}$(2)-Er1- $Pn^{\dagger}_{cent}$(4)		140.25(7)
Fold angle ($Pn^{\dagger}_{1}$-$Pn^{\dagger}_{2}$)		24.41
Fold angle ($Pn^{\dagger}_{3}$-$Pn^{\dagger}_{4}$)		25.14

* The fold angle is defined as the dihedral angle between the two C_5 planes of pentalene ligand.

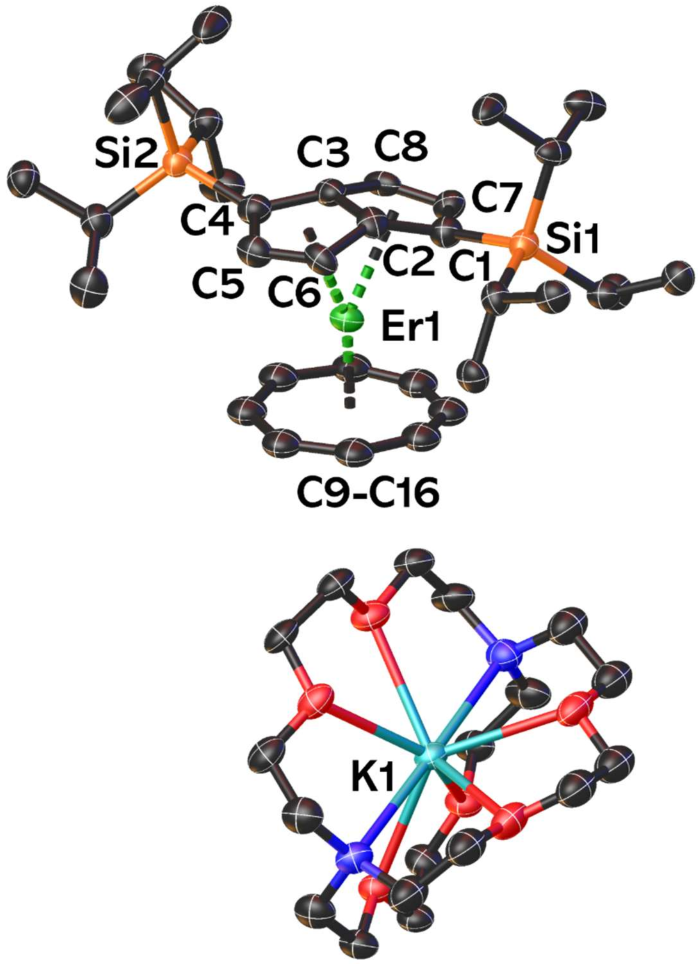

Figure S4. Thermal ellipsoid representation (30% probability) of the structure of [K(2.2.2-crypt)][**2**]. For clarity, hydrogen atoms are not shown.

Table S3. Selected distances and angles for [K(2.2.2-crypt)][**2**].

	**Length/Å**	**Angle/°**
Er1-C1	2.678(9)	
Er1-C2 body	2.386(7)	
Er1-C3 body	2.374(8)	
Er1-C4	2.682(9)	
Er1-C5 tip	2.791(8)	
Er1-C6	2.675(8)	
Er1-C7 tip	2.799(10)	
Er1-C8	2.632 (8)	
C2(br)-C3(br)	1.423(16)	
Er1-C9(COT)	2.608(12)	
Er1-C10(COT)	2.582(11)	
Er1-C11(COT)	2.556(11)	
Er1-C12(COT)	2.582(9)	
Er1-C13(COT)	2.633(10)	
Er1-C14(COT)	2.657(10)	
Er1-C15(COT)	2.568(11)	
Er1-C16(COT)	2.590(12)	
Er1-COT_{cent}	1.863(3)	
Er1-$Pn^{\dagger}_{cent}$(1)	2.278(4)	
Er1-$Pn^{\dagger}_{cent}$(2)	2.288(3)	
$Pn^{\dagger}_{cent}$(1)-Er1-COT_{cent}		152.06(17)
$Pn^{\dagger}_{cent}$(2)-Er1-COT_{cent}		158.25(19)
(C2-C3)-Er1-COT_{cent}		172.1(7)
Fold angle* ($Pn^{\dagger}_1$-$Pn^{\dagger}_2$)		23.06

* The fold angle is defined as the dihedral angle between the two C_5 planes of pentalene ligand.

Magnetic measurements

Magnetic measurements were performed on a Quantum Design MPMS3 SQUID magnetometer equipped with a 7 T magnet. Samples were prepared by crushing the crystalline materials before transferring them to a 7 mm NMR tube and covering them in eicosane. Then the tubes were flame sealed under a static vacuum. The eicosane was melted in a warm water bath at 40 °C to prevent crystallite torquing. Diamagnetic corrections were performed using Pascal's constants.[7]

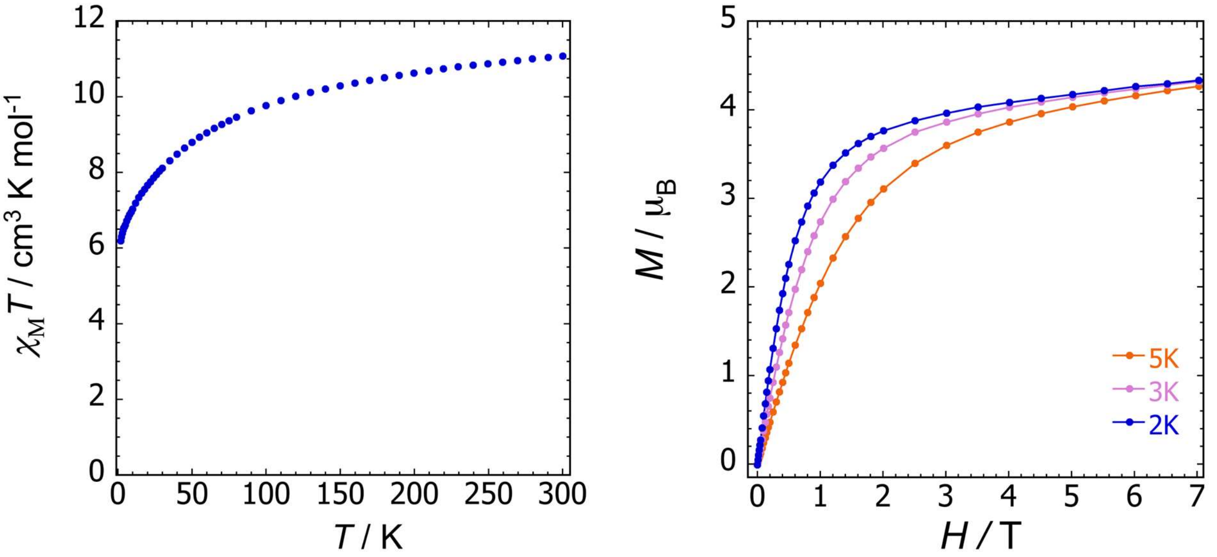

Figure S5. Temperature dependence of the molar magnetic susceptibility (left) and isothermal field-dependent magnetization (right) plot for [K(2.2.2-cryptand)][**1**]. The value of $\chi_M T$ at 300 K is 11.07 cm^3 K mol^{-1} and 6.19 cm^3 K mol^{-1} at 2 K. The value of M_{sat} at 2.0 K and 7 T is 4.33 μ_B.

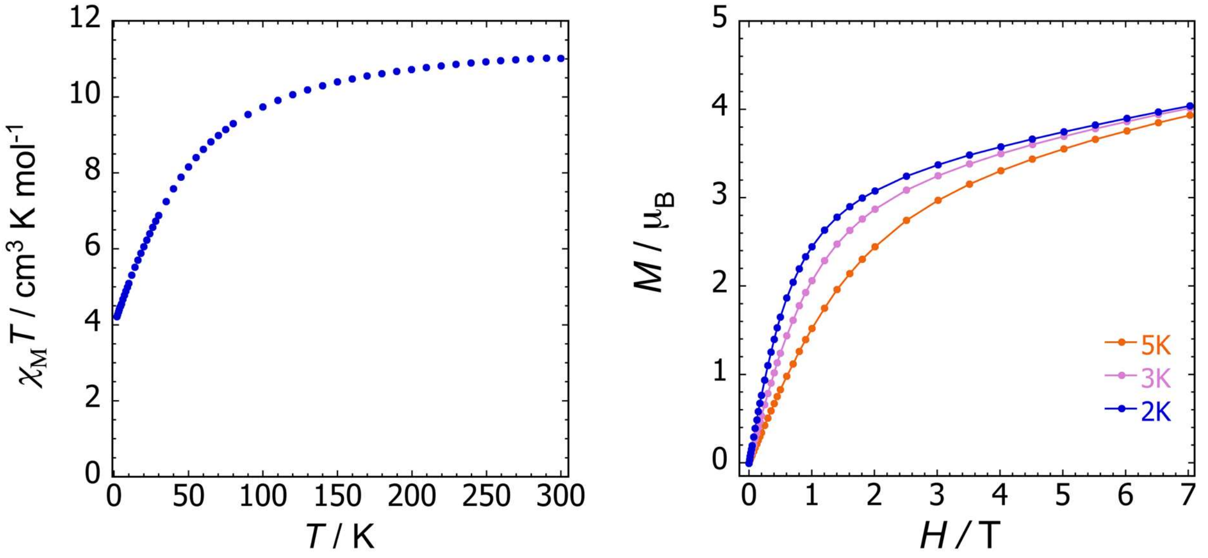

Figure S6. Temperature dependence of the molar magnetic susceptibility (left) and isothermal field-dependent magnetization (right) for K(2.2.2-cryptand)][**2**]. The value of $\chi_M T$ is 11.01 cm^3 K mol^{-1} at 300 K and 4.21 cm^3 K mol^{-1} at 2 K. The value of M_{sat} at 2.0 K and 7 T is 4.04 μ_B.

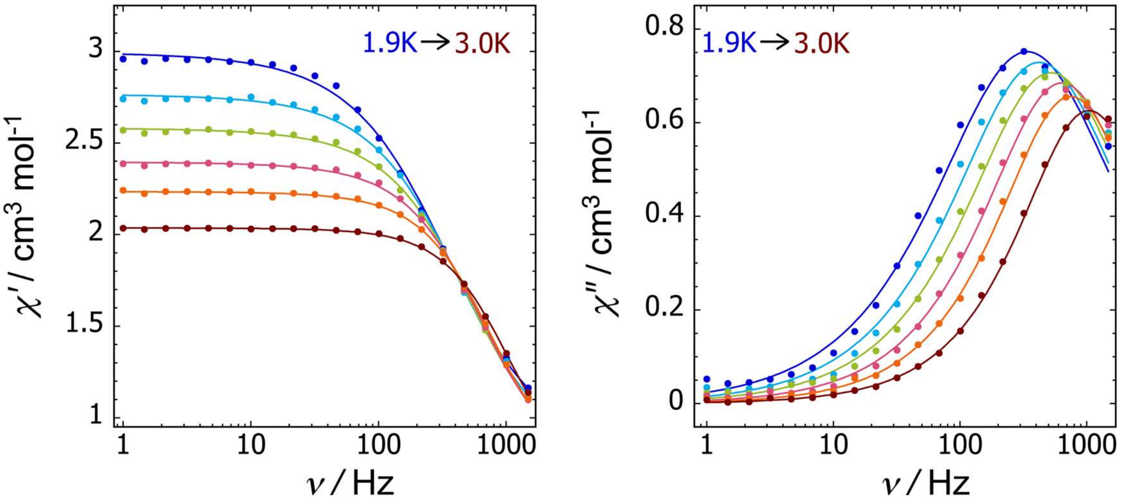

Figure S7. Left: Frequency dependence of the in-phase susceptibility (χ') and right: out-of-phase susceptibility (χ'') for K(2.2.2-cryptand)][**1**] measured under an applied DC field of 600 Oe and at ν = 1 to 1488 Hz and temperatures of 1.9-3.0 K.

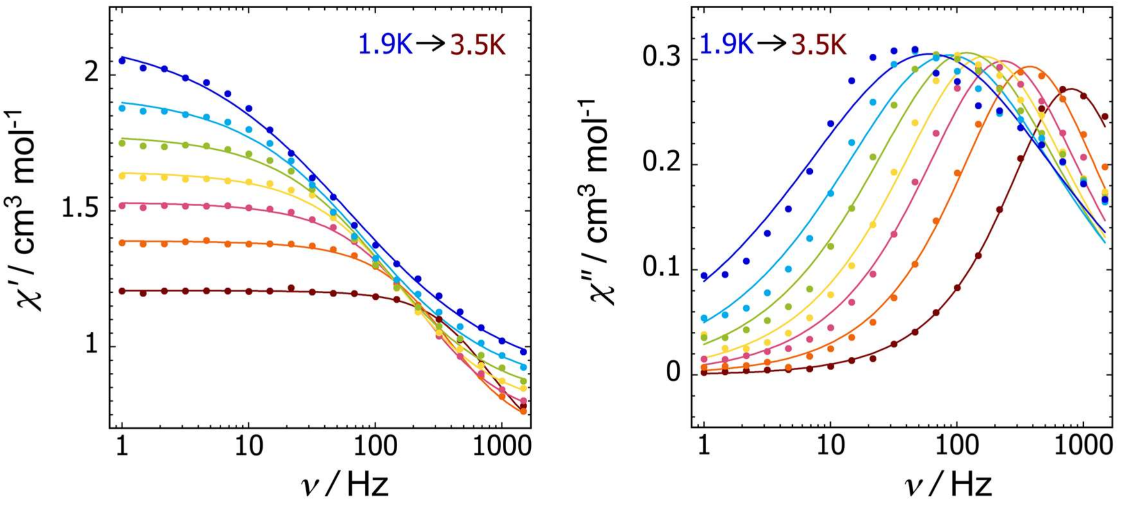

Figure S8. Left: frequency dependence of the in-phase susceptibility (χ'). Right: out-of-phase susceptibility (χ'') for K(2.2.2-cryptand)][**2**] measured under an applied DC field of 600 Oe and at ν = 1 to 1488 Hz and temperatures of 1.9-3.5 K.

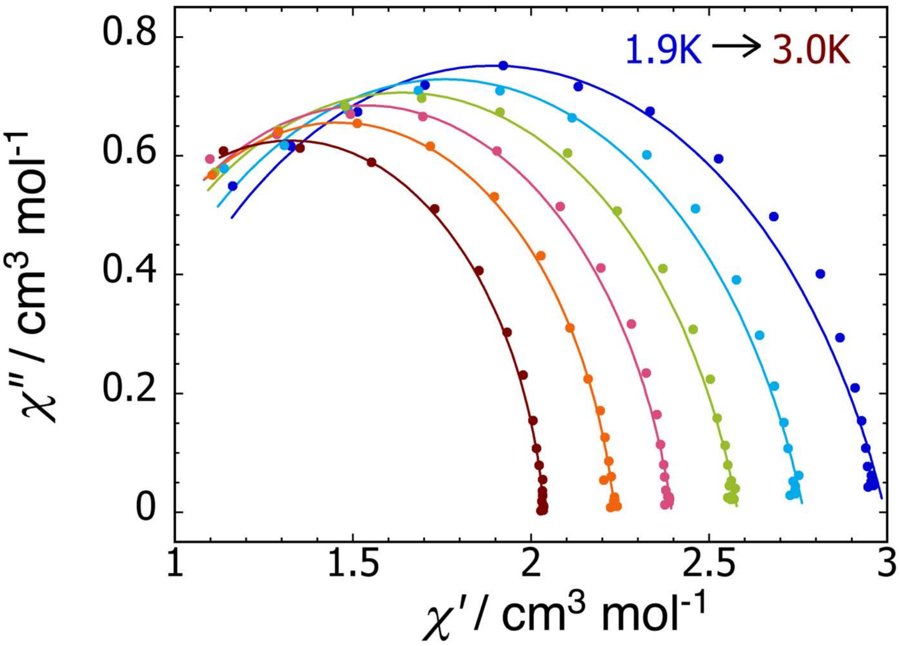

Figure S9. Cole-Cole plots for the AC susceptibilities under an applied DC field of 600 Oe for K(2.2.2-cryptand)][**1**] from 1.9-3.0 K. Solid lines represent fits to the data using equations S1 and S2.

$$\chi'(\nu_{ac}) = \chi_S + \frac{(\chi_T - \chi_S)[1 + (2\pi\nu_{ac}\tau)^{(1-\alpha)} Sin(\frac{\alpha\pi}{2})]}{1 + 2(2\pi\nu_{ac}\tau)^{(1-\alpha)} Sin\left(\frac{\alpha\pi}{2}\right) + (2\pi\nu_{ac}\tau)^{2(1-\alpha)}} \qquad \text{(eqn. S1)}$$

$$\chi''(\nu_{ac}) = \frac{(\chi_T - \chi_S)(2\pi\nu_{ac}\tau)^{(1-\alpha)} Cos(\frac{\alpha\pi}{2})}{1 + 2(2\pi\nu_{ac}\tau)^{(1-\alpha)} Sin\left(\frac{\alpha\pi}{2}\right) + (2\pi\nu_{ac}\tau)^{2(1-\alpha)}} \qquad \text{eqn. S2)}$$

Table S4. Relaxation fitting parameters for K(2.2.2-cryptand)][**1**] corresponding to Figure S9.

***T* (K)**	**χ_S (cm^{3}mol^{-1})**	**χ_T (cm^{3}mol^{-1})**	α	**τ (s)**
1.8998	0.794	3.00	0.237	0.000468
2.0999	0.754	2.77	0.202	0.000376
2.3000	0.695	2.58	0.181	0.000304
2.4999	0.676	2.40	0.144	0.000249
2.7000	0.686	2.24	0.105	0.000212
2.9999	0.616	2.04	0.0806	0.000153

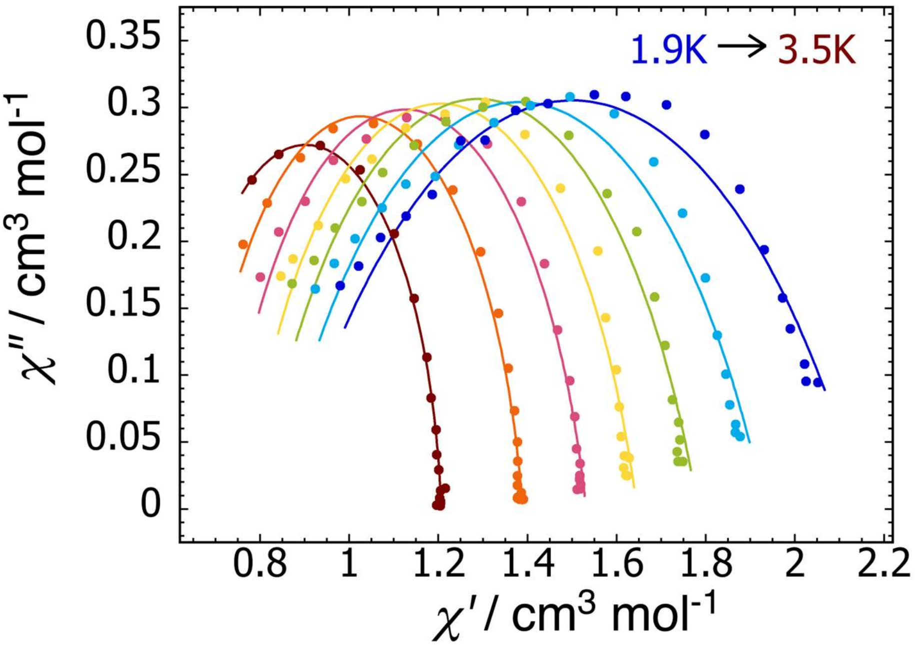

Figure S10. Cole-Cole plots for the AC susceptibilities under an applied DC field of 600 Oe for K(2.2.2-cryptand)][**2**] from 1.9-3.5 K. Solid lines represent fits to the data using equations S1 and S2.

Table S5. Relaxation fitting parameters for K(2.2.2-cryptand)][**2**] corresponding to Figure S10.

T **(K)**	χ_S **(cm^3mol^{-1})**	χ_T **(cm^3mol^{-1})**	α	τ **(s)**
1.8968	0.847	2.15	0.443	0.00262
2.0998	0.830	1.93	0.358	0.00183
2.3001	0.795	1.78	0.292	0.00133
2.5000	0.764	1.65	0.233	0.000960
2.6999	0.716	1.53	0.195	0.000687
2.9999	0.662	1.39	0.135	0.000422
3.4994	0.600	1.21	0.0686	0.000198

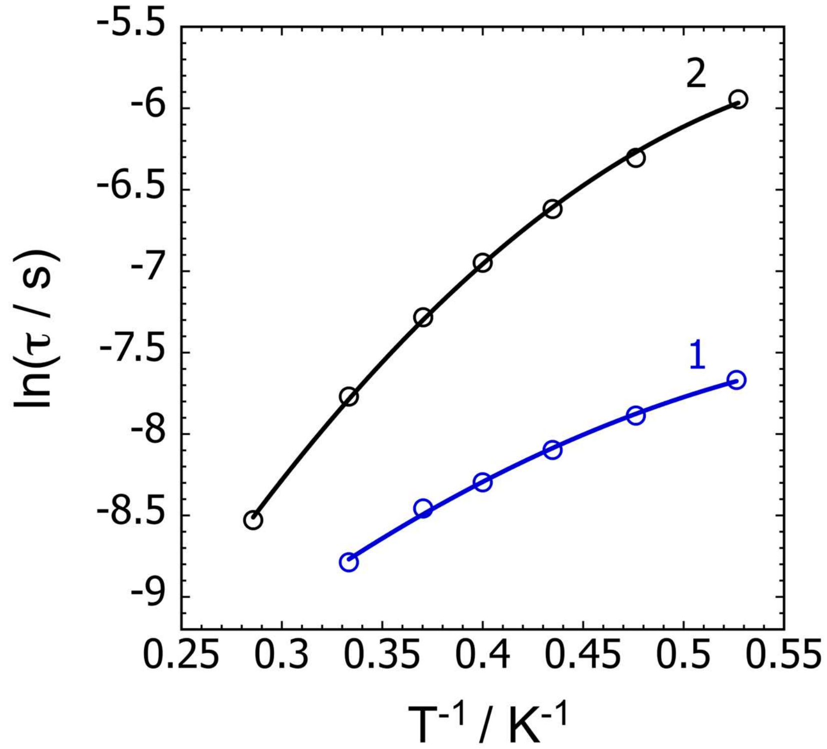

Figure S11. Plot of $\ln(\tau/s)$ vs. T^{-1} for K(2.2.2-cryptand)][**1**] and K(2.2.2-cryptand)][**2**]. Circles are experimental data points and solid line is the best fit considering Raman and QTM processes using $\tau^{-1} = CT^n + \tau_{\text{QTM}}^{-1}$.

Magnetic relaxation fitting parameters for K(2.2.2-cryptand)][**2**]: C = 8.44 ± 0.927 s^{-1} K^{-n}, n = 5.07 ± 0.094, and τ_{QTM} = 5.8 ± 0. 135 ms.

Magnetic relaxation fitting parameters for K(2.2.2-cryptand)][**1**]: C = 179 ± 74.61 s^{-1} K^{-n}, n = 3.13 ± 0.34, and τ_{QTM} = 1.2 ± 0. 281 ms.

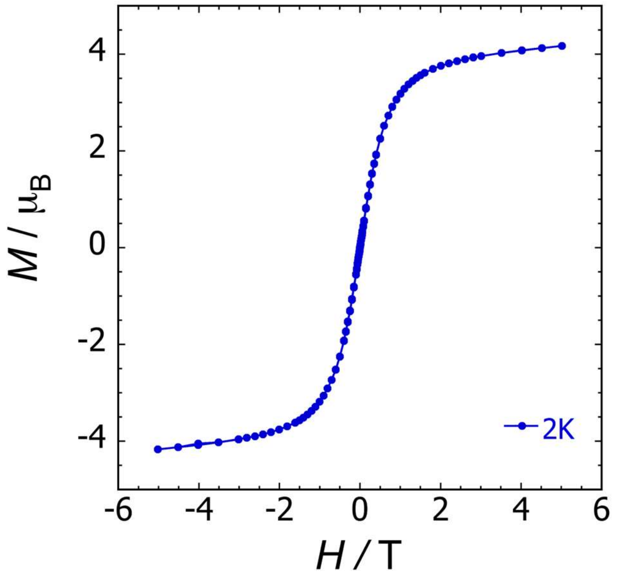

Figure S12. Magnetic hysteresis data for K(2.2.2-cryptand)][**1**] at 2K. The following field sweep rates were used: (0.25 mT s^{-1} |0-0.02| T; 0.45 mT s^{-1} |0.02-0.06| T; 0.95 mT s^{-1} |0.06-0.1| T; 2.27 mT s^{-1} |0.1-0.4| T; 4 mT s^{-1} |0.4-1.6| T; 6.66 mT s^{-1} |1.6-3| T; 11.1 mT s^{-1} |3-5.0| T).

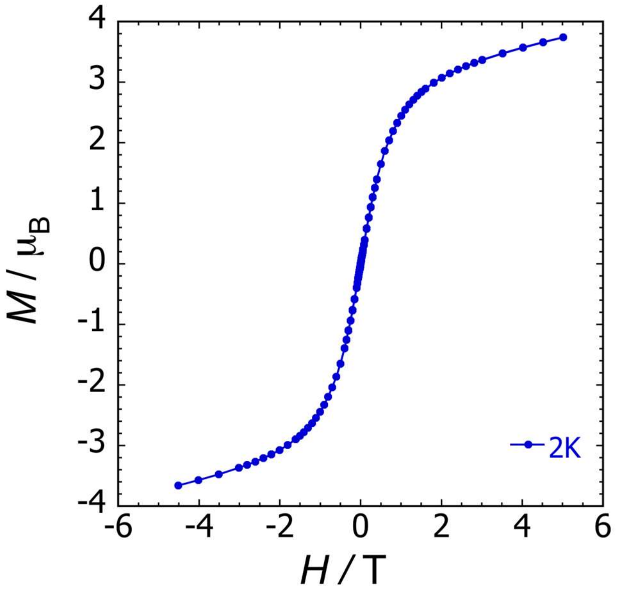

Figure S13. Magnetic hysteresis data for K(2.2.2-cryptand)][**2**] at 2K. The following field sweep rates were used: (0.25 mT s^{-1} |0-0.02| T; 0.45 mT s^{-1} |0.02-0.06| T; 0.95 mT s^{-1} |0.06-0.1| T; 2.17 mT s^{-1} |0.1-0.4| T; 4 mT s^{-1} |0.4-1.6| T; 6.66 mT s^{-1} |1.6-3| T; 11.1 mT s^{-1} |3-5.0| T).

Computational details

All the calculations were carried out on the coordinates obtained from the X-ray structure using ORCA 6.0.0 software package.[8] The position of hydrogen atoms was optimized at the DFT level using pure GGA PBE exchange correlation functional[9-10] keeping constant the position of other atoms. The calculations were of the CASSCF/QDPT/SINGLE_ANISO type and the DKH (Douglas-Kroll-Hess) Hamiltonian was used throughout to account for relativistic effects. We employed the SARC2-DKH-QZVP basis set for Er(III) whereas all other atoms were treated with the DKH-def2-TZVP basis set in combination with 'AutoAux' auxiliary basis set.[11-13] In the active space, we have considered 11 electrons in 7 f-orbital CAS (7,11) for both complexes. Further, 35 quartet and 90 doublet states for were solved in the state-averaged (SA) calculations. To consider the spin-orbit coupling, we have also used the quasi-degenerate perturbation theory (QDPT) approach using SA-CASSCF wave functions.[14] The SINGLE_ANISO module[15] as implemented in ORCA has been used to compute the g-tensors and crystal field parameters of the low-lying excited state using previously calculated spin-orbit states.

Table S6. Computed energy of the KDs, g-tensors and wavefunction compositions for K(2.2.2-cryptand)][**1**].

KD	E / (cm^{-1})	g_x	g_y	g_z	Wavefunction composition
1	0.000	0.934	4.295	13.682	51.8%|±15/2>+0.60%|±13/2>+25.80%|±11/2>+2.40%|±9/2>+4.90%|±7/2>+2.70%|±5/2>+3.80%|±3/2>+3.90%|±1/2>
2	45.063	1.557	4.170	10.967	10.70%|±15/2>+1.30%|±13/2>+0.70%|±11/2>+29.30%|±9/2>+20.0%|±7/2>+16.30%|±5/2>+12.90%|±3/2>+8.70%|±1/2>
3	89.165	4.552	5.957	7.831	1.10%|±15/2>+11.90%|±13/2>+6.60%|±11/2>+28.50%|±9/2>+34.20%|±7/2>+14.10%|±5/2>+2.60%|±3/2>+1.0%|±1/2>
4	219.841	1.304	3.091	10.947	0.70%|±15/2>+0.80%|±13/2>+2.0%|±11/2>+9.0%|±9/2>+29.0%|±7/2>+41.50%|±5/2>+2.80%|±3/2>+14.10%|±1/2>
5	295.382	1.565	3.991	8.851	1.70%|±15/2>+3.90%|±13/2>+1.30%|±11/2>+10.90%|±9/2>+7.50%|±7/2>+16.40%|±5/2>+51.10%|±3/2>+7.30%|±1/2>
6	376.489	0.225	1.793	12.040	10.60%|±15/2>+24.90%|±13/2>+15.90%|±11/2>+7.10%|±9/2>+4.30%|±5/2>+10.30%|±3/2>+26.80%|±1/2>
7	427.122	0.174	2.000	9.665	13.70%|±15/2>+40.70%|±13/2>+32.90%|±11/2>+9.0%|±9/2>+1.60%|±7/2>+0.50%|±5/2>+1.30%|±3/2>+0.20%|±1/2>
8	497.391	0.054	0.176	13.727	5.40%|±15/2>+16.0%|±13/2>+14.80%|±11/2>+3.60%|±9/2>+2.70%|±7/2>+4.20%|±5/2>+15.20%|±3/2>+37.90%|±1/2>

Table S7. Computed energy of the KDs, g-tensors and wavefunction compositions for K(2.2.2-cryptand)][**2**].

<table>
<tr><th>KD</th><th>E / (cm^{-1})</th><th>g_x</th><th>g_y</th><th>g_z</th><th>Wavefunction composition</th></tr>
<tr><td>1</td><td>0.000</td><td>1.649</td><td>1.919</td><td>12.75</td><td>51.8%|±15/2>+0.70%|±13/2>+20.20%|±11/2>+6.20%|±9/2>+6.60%|±7/2>+5.30%|±5/2>+4.80%|±3/2>+4.50%|±1/2></td></tr>
<tr><td>2</td><td>24.659</td><td>0.369</td><td>2.155</td><td>13.11</td><td>8.0%|±15/2>+2.20%|±13/2>+5.20%|±11/2>+11.50%|±9/2>+21.80%|±7/2>+13.10%|±5/2>+19.80%|±3/2>+18.30%|±1/2></td></tr>
<tr><td>3</td><td>78.349</td><td>3.228</td><td>4.822</td><td>9.226</td><td>2.40%|±15/2>+11.40%|±13/2>+2.50%|±11/2>+16.30%|±9/2>+14.60%|±7/2>+26.10%|±5/2>+18.70%|±3/2>+7.20%|±1/2></td></tr>
<tr><td>4</td><td>96.911</td><td>0.110</td><td>1.015</td><td>12.196</td><td>1.50%|±15/2>+3.90%|±13/2>+4.50%|±11/2>+10.90%|±9/2>+11.0%|±7/2>+27.30%|±5/2>+17.10%|±3/2>+23.70%|±1/2></td></tr>
<tr><td>5</td><td>221.315</td><td>0.290</td><td>2.042</td><td>11.183</td><td>8.50%|±15/2>+20.30%|±13/2>+4.0%|±11/2>+12.30%|±9/2>+27.50%|±7/2>+5.40%|±5/2>+15.10%|±3/2>+6.90%|±1/2></td></tr>
<tr><td>6</td><td>256.180</td><td>0.535</td><td>1.119</td><td>12.25</td><td>14.10%|±15/2>+13.50%|±13/2>+9.30%|±11/2>+25.30%|±9/2>+9.70%|±7/2>+3.20%|±5/2>+4.20%|±3/2>+20.90%|±1/2></td></tr>
<tr><td>7</td><td>374.175</td><td>0.178</td><td>0.482</td><td>15.81</td><td>5.70%|±15/2>+24.20%|±13/2>+26.40%|±11/2>+9.90%|±9/2>+3.60%|±7/2>+9.50%|±5/2>+12.20%|±3/2>+8.30%|±1/2></td></tr>
<tr><td>8</td><td>418.990</td><td>0.239</td><td>0.368</td><td>16.05</td><td>7.80%|±15/2>+23.70%|±13/2>+27.90%|±11/2>+7.70%|±9/2>+5.20%|±7/2>+9.0%|±5/2>+8.30%|±3/2>+10.30%|±1/2></td></tr>
</table>

Table S8. Calculated crystal field parameters for K(2.2.2-cryptand)][**1**] and K(2.2.2-cryptand)][**2**].

k	*q*	K(2.2.2-cryptand)][**1**]	K(2.2.2-cryptand)][**2**]
	-2	-0.8329E+00	0.4578E+00
	-1	-0.1075E+01	0.1803E+01
2	0	-0.2302E+00	0.2142E+00
	1	-0.1531E+00	0.1188E+01
	2	0.2126E+00	-0.1849E+00
	-4	0.8429E-03	-0.2512E-01
	-3	-0.1492E-01	-0.1398E-02
	-2	-0.3579E-03	-0.1941E-01
	-1	-0.1240E-01	-0.5817E-02
4	0	-0.1729E-03	-0.3279E-02
	1	0.3583E-03	-0.1237E-01
	2	0.1662E-01	-0.9027E-02
	3	-0.1228E-02	-0.3460E-02
	4	-0.1105E-01	0.2278E-01
	-6	-0.3781E-04	0.3058E-04
	-5	-0.9315E-03	-0.1143E-02
	-4	-0.1609E-04	-0.3109E-03
	-3	-0.7893E-03	0.2033E-03
	-2	-0.2286E-04	-0.3873E-03
	-1	0.2438E-03	0.1107E-03
6	0	-0.8759E-04	-0.4159E-04
	1	0.2991E-04	0.1024E-03
	2	0.6419E-03	-0.2273E-03
	3	-0.6030E-04	-0.1930E-03
	4	-0.7778E-04	0.1517E-03
	5	-0.2243E-03	0.2375E-02
	6	0.6964E-03	0.2647E-03

Table S9. Calculated transition magnetic moment matrix elements (in Bohr magneton) for K(2.2.2-cryptand)][**1**].

Climbing Transition			Crossing Transition		
Initial KD	Final KD	Magnitude	Initial KD	Final KD	Magnitude
1	2	1.573	1	1	0.8716
1	3	1.521	1	2	1.400
1	4	0.5380	1	3	0.7476
1	5	0.3135	1	4	0.3602
1	6	0.4806	1	5	0.1325
1	7	0.7285	1	6	0.6583
1	8	0.2576	1	7	0.3096
2	3	1.676	1	8	0.2039
2	4	0.9248	2	2	1.738
2	5	0.8100	2	3	0.9416
2	6	0.2722	2	4	0.9150
2	7	0.7699	2	5	0.7439
2	8	0.2243	2	6	0.7379
3	4	0.9578	2	7	0.3339
3	5	0.6886	2	8	0.1386
3	6	1.343	3	3	1.835
3	7	0.3770	3	4	1.155
3	8	0.1702	3	5	1.121
4	5	2.481	3	6	0.3921
4	6	0.1226	3	7	0.9172
4	7	0.7330	3	8	0.1325
4	8	0.5737	4	4	2.325
5	6	0.9179	4	5	0.9233
5	7	0.2298	4	6	0.1894
5	8	1.393	4	7	0.2959
6	7	0.8427	4	8	0.6108
6	8	0.6849	5	5	2.110
7	8	0.8912	5	6	0.3406
			5	7	0.3746
			5	8	1.198
			6	6	2.048
			6	7	2.263
			6	8	0.5523
			7	7	1.324
			7	8	1.607
			8	8	0.3429

Table S10. Calculated transition magnetic moment matrix elements (in Bohr magneton) for K(2.2.2-cryptand)][**2**].

Climbing Transition			Crossing Transition		
Initial KD	Final KD	Magnitude	Initial KD	Final KD	Magnitude
1	2	1.141	1	1	0.594
1	3	1.757	1	2	2.094
1	4	1.326	1	3	0.578
1	5	1.318	1	4	0.7715
1	6	0.899	1	5	0.4755
1	7	0.3774	1	6	0.2110
1	8	0.133	1	7	0.2501
2	3	1.545	1	8	0.0692
2	4	1.049	2	2	1.249
2	5	1.090	2	3	0.9655
2	6	0.8242	2	4	0.4563
2	7	0.7837	2	5	0.6570
2	8	0.1140	2	6	0.8963
3	4	1.536	2	7	0.2168
3	5	0.7824	2	8	0.1889
3	6	1.437	3	3	1.955
3	7	0.358	3	4	1.981
3	8	0.6029	3	5	1.029
4	5	1.208	3	6	0.4965
4	6	0.8806	3	7	0.311
4	7	0.375	3	8	0.3022
4	8	0.4130	4	4	2.109
5	6	1.109	4	5	1.339
5	7	2.401	4	6	1.044
5	8	0.488	4	7	0.1318
6	7	0.5868	4	8	0.6869
6	8	2.348	5	5	0.4576
7	8	0.222	5	6	1.088
			5	7	0.2073
			5	8	0.6590
			6	6	0.382
			6	7	0.829
			6	8	0.1977
			7	7	0.136
			7	8	0.580
			8	8	0.1274

References

1 F. G. N. Cloke, M. C. Kuchta, R. M. Harker, P. B. Hitchcock, J. S. Parry, *Organometallics* **2000**, *19*, 5795-5798.

2 S. M. Cendrowski-Guillaume, G. Le Gland, M. Nierlich, M. Ephritikhine, *Organometallics* **2000**, *19*, 5654-5660.

3 S. M. Cendrowski-Guillaume, M. Nierlich, M. Lance, M. Ephritikhine, *Organometallics* **1998**, *17*, 786-788.

4 O. V. Dolomanov, L. J. Bourhis, R. J. Gildea, J. A. K. Howard, H. Puschmann, *J. Appl. Crystallogr.* **2009**, *42*, 339-341.

5 L. J. Bourhis, O. V. Dolomanov, R. J. Gildea, J. A. K. Howard, H. Puschmann, *Acta Crystallogr. Sect. A* **2015**, *71*, 59-75.

6 G. Sheldrick, *Acta Crystallogr. Sect. C* **2015**, *71*, 3-8.

7 G. A. Bain, J. F. Berry, *J. Chem Ed.* **2008**, *85*, 532.

8 F. Neese, *WIREs Comput. Mol. Sci.* **2022**, *12*, e1606.

9 J. P. Perdew, K. Burke, M. Ernzerhof, *Phys. Rev. Lett.* **1997**, *78*, 1396-1396.

10 J. P. Perdew, K. Burke, M. Ernzerhof, *Phys. Rev. Lett.* **1996**, *77*, 3865-3868.

11 D. Aravena, F. Neese, D. A. Pantazis, *J. Chem. Theor. Comput.* **2016**, *12*, 1148-1156.

12 J. Chmela, M. E. Harding, *Mol. Phys.* **2018**, *116*, 1523-1538

13 F. Weigend, R. Ahlrichs, *Phys, Chem. Chem. Phys.* **2005**, *7*, 3297-3305.

14 D. Ganyushin, F. Neese, *J. Chem. Phys.* **2006**, *125*, 024103.

15 L. F. Chibotaru, L. Ungur, *J. Chem. Phys.* **2012**, *137*, 064112.
